# Italian Wines

**Published:** 1907-06-29

**Authors:** 


ITALIAN WINES.

We have received a letter from Mr. Egidio Vitali, of 5 and 6 Great Winchester Street, E.C., on the subject of Italian wines, and regret we have no space for the whole of it In effect Mr. Vitali challenges our Commission's statement that Italian wines are unsuitable fcr consumption at meals, and, owing to the high percentage of alcohol they contain, cannot properly be called light wines. He then states that the every-day drink of the people in Italy is a very light red wine resembling the *vin ordinaire* of France, and adds that Chianti is the Italian wine for which there is most demand in this country, and that the quantity of alcohol it contains varies from sixteen to eighteen per cent. Nearly all the Italian wines shipped to this country, Mr. Vitali claims, are light wines with an alcoholic strength of from sixteen to twenty per cent. He claims that Italian wines are not fortified for export to England and are therefore ideal wines for consumption at meals.

.·. While we are indebted to Mr. Vitali for his interesting communication we are obliged to say that his letter is scarcely pertinent to the remarks which were made concerning Italian wines. We did not discuss Italian wines as they are consumed in Italy, we merely referred to the wines which are consumed in this country, and as a matter of fact if Mr. Vitali will carefully read what we did say he will see that we did not make the statement to which he demurs. There can be no doubt, however, that, taken on the whole—we cannot, of course, enter into any discussion on the merits of a particular brand—the Italian wines are decidedly more alcoholic than the light French wines.—Ed. The Hospital.

